# L-type calcium channel-dependent inhibitory plasticity in the thalamus

**DOI:** 10.1152/jn.00918.2013

**Published:** 2014-03-12

**Authors:** Sarah R. Hulme, William M. Connelly

**Affiliations:** ^1^School of Physiology and Pharmacology, School of Medical Sciences, Bristol, United Kingdom; and; ^2^Neuroscience Division, Cardiff School of Biosciences, Cardiff, United Kingdom

**Keywords:** thalamus, synaptic plasticity, LTP, GABA, NOS, L-type calcium

## Abstract

Thalamocortical neurons integrate sensory and cortical activity and are regulated by input from inhibitory neurons in the thalamic reticular nucleus. Evidence suggests that during bursts of action potentials, dendritic calcium transients are seen throughout the dendritic tree of thalamocortical cells. Here, we review a recent study that suggests these calcium transients regulate inhibitory input, and we attempt to reconcile studies that differ on which ion channels are the source of the calcium.

the thalamus is a critical relay and processing station for a wide variety of neural signals. Incoming sensory information, as well as descending cortical input, drives excitation of thalamocortical (TC) cells which then project to the cortex and other regions. The activity of excitatory thalamic neurons, and therefore the relay of sensory information to the cortex, is dynamically regulated by GABAergic inhibition. For large parts of the thalamus, the primary source of this inhibition is the thalamic reticular nucleus (TRN). While developmental plasticity of thalamic synapses has been well studied (e.g., Hooks and Chen 2006), activity-dependent plasticity of the adult thalamus has received comparatively little attention.

Most thalamic neurons produce bursts of action potentials when depolarized from relatively hyperpolarized potentials. These bursts are riding on a sustained depolarizing envelope which is called a low-threshold calcium potential (LTCP) and is mediated by T-type calcium channels. In contrast, when thalamic neurons are relatively depolarized, T-type calcium channels are inactivated and hence cannot cause neurons to fire bursts. Therefore, the firing behavior of thalamic neurons strongly depends on the membrane potential they are resting at: when they are more hyperpolarized, they generate LTCPs and bursts, and when they are more depolarized, they typically fire individual action potentials. Bursts have previously been demonstrated to cause a large calcium transient throughout the somatodendritic extent of thalamocortical neurons (Errington et al. 2010); however, a cellular function for these large calcium signals has not been found.

Sieber et al. (2013) performed whole cell recordings in the posteromedial complex (PoM) of the thalamus in rat brain slices. In the presence of ionotropic glutamate receptor antagonists, they used extracellular stimulation to activate axons presumably from the TRN and thereby evoked inhibitory postsynaptic potentials (IPSPs). These IPSPs comprised an early component, consistent with a GABA_A_-mediated response, as well as a profound late component, which was insensitive to gabazine and had a reversal potential of −80 mV, and therefore assumed to be GABA_B_ receptor mediated.

When cells were held at approximately −60 mV, 60 pairings at 0.1 Hz of evoked IPSPs with two current injection-induced action potentials (single action potentials per depolarization; 10 ms between depolarizations) produced an ∼50% potentiation of IPSP amplitude, which lasted for the length of the recording (inhibitory long-term potentiation; iLTP). Somewhat surprisingly, the relative timing of the IPSP and the pair of action potentials did not affect the magnitude of the potentiation, and, importantly, postsynaptic spiking alone was sufficient to induce iLTP. These results demonstrated that the iLTP was not a type of spike-timing-dependent plasticity and that the induction was solely dependent on postsynaptic activity. Additionally, iLTP was dependent on the frequency of pairings; a conserved number of postsynaptic spikes but at higher frequencies resulted in less (1 Hz) or no (5 Hz) iLTP.

The observed potentiation required a rise in postsynaptic intracellular Ca^2+^ concentration as intracellular infusions of the calcium chelator BAPTA prevented iLTP induction. For this reason, Sieber et al. (2013) subsequently utilized two-photon calcium imaging to examine whether LTCPs were playing a critical role in the induction of the iLTP as well as to potentially illuminate the role of different calcium channel types in iLTP.

When two suprathreshold depolarizations were delivered from hyperpolarized potentials (approximately −60 and −70 mV), a broad depolarizing envelope and multiple action potentials were generated, characterizing these responses as bursts riding on a LTCP. Consistent with this, concurrent two-photon imaging revealed global dendritic rises in calcium concentration across the dendritic tree. In contrast, from a more depolarized potential of approximately −50 mV, suprathreshold depolarization only generated single action potentials and a smaller resultant dendritic calcium signal that decremented along the dendritic tree. The use of the voltage-gated sodium channel antagonist TTX showed that the rises in calcium concentration were largely dependent on sodium channel function when the action potentials were generated from −50 mV, but largely independent of sodium channels when generated from −60 or −70 mV. At these more hyperpolarized potentials, combined application of TTX and the L-type calcium channel antagonist nimodipine produced a further reduction in the amplitude of the calcium transients by ∼30% and 50% for −70 mV and −60 mV, respectively, showing that the calcium transients were partially dependent on L-type calcium channels. Importantly, the potentiation induced by pairing IPSPs and action potentials (from −60 mV) was completely prevented by application of nimodipine, suggesting the L-type channel-mediated component of the total calcium transient was essential for the induction of iLTP. In accordance with this, and the relative contribution of L-type calcium channels to the calcium transient elicited by suprathreshold stimulation from different membrane potentials, iLTP was also induced when pairing was delivered from −70 mV but not from −50 mV.

The pairing-induced potentiation appeared to be expressed presynaptically, as both the GABA_A_ and GABA_B_ receptor-mediated components were equally potentiated, and the iLTP altered the paired pulse ratio as well as the variance of IPSPs in a manner consistent with presynaptic modulation. Given the exclusively postsynaptic induction requirements and presynaptic expression, a retrograde messenger was considered likely to be a key component of iLTP induction. In line with this expectation, bath application of the nitric oxide donor SNAP potentiated IPSPs to a similar extent as the pairing protocol, while pairing was unable to potentiate IPSPs in the presence of the NO scavenger cPTIO or ODQ-mediated inhibition of a downstream target of NO, guanylyl cyclase.

The findings of Sieber et al. (2013) demonstrate that inhibitory inputs (likely from TRN) to excitatory PoM neurons can be potentiated in response to rises in postsynaptic calcium. This calcium influx is partially dependent on L-type calcium channels, and the influx through these channels is critical for iLTP induction. On the surface, the results of Sieber et al. appear to be at odds with those of Errington et al. (2010), who reported that the rise in calcium in TC dendrites during LTCPs was completely abolished by T-type channel antagonists. However, these results are in fact easily married by the notion that the depolarizing drive that mediates LTCP is conducted through T-type channels, which then depolarize dendrites enough to open L-type channels (Fig. 1). This is alluded to by Sieber et al., and, consistent with this, they note that the rising phase of the calcium transient was not altered by L-type channel antagonism. While Sieber et al. did not explicitly examine the role of T-type channels in the iLTP, it would follow that antagonism of these channels in their preparation would also prevent the induction of iLTP through preventing the subsequent L-type channel activation. The example traces of Sieber et al. also appear to indicate an increased calcium transient amplitude with dendritic distance. However, as dye loading is often inhomogeneous in fine dendrites, this conclusion may be unwarranted. As lower concentrations of calcium indicator provide weaker Ca^2+^ buffering, they allowed larger calcium transients. Indeed, the results presented suggest a faster decaying fluorescent signal in distal dendrites, which is further evidence of weaker Ca^2+^ buffering in the distal dendrites (although this may be due to unrepresentative examples, as the kinetics of the fluorescent transient are not reported quantitatively). Seiber et al. needed to report the time integral of the fluorescent signal against distance to draw conclusions about the magnitude of the calcium transient (Helmchen et al. 1996).

**Fig. 1.**
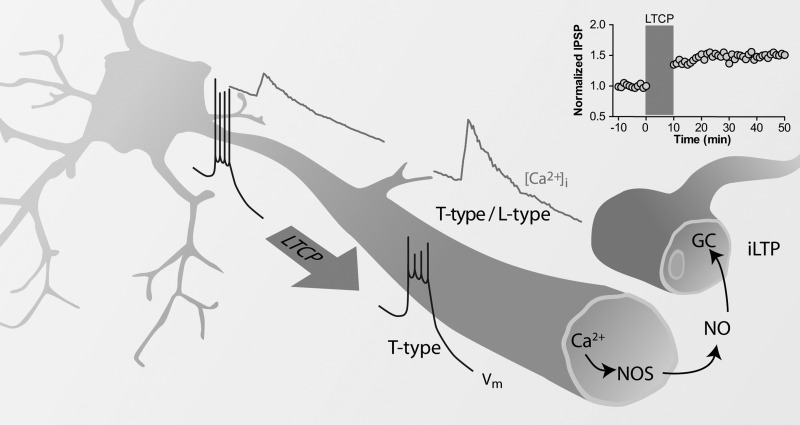
Schematic showing the proposed mechanism of inhibitory long-term potentiation (iLTP) recruited by thalamocortical cell bursting. Somatic current injection produces a T-type calcium channel-mediated, low-threshold calcium potential and burst of action potentials that propagates into dendritic compartments. In the dendrite, the T-type-driven depolarization opens L-type calcium channels, leading to further calcium influx. The rise in calcium activates NOS, releasing NO, which acts as a retrograde transmitter, enhancing the activity of guanylyl cyclase and changing the presynaptic release probability.

The fact that L-type calcium channel antagonists were able to completely abolish iLTP while only reducing the peak calcium transient by 50% led Sieber et al. (2013) to conclude that the calcium entering via L-type channels is somehow in a privileged position to trigger plasticity. While this indeed may be the case, there are alternative explanations as well as some further questions. It may instead be that there is a threshold amount of calcium entry needed to trigger iLTP, and that by blocking L-type channels, Sieber et al. has pushed the calcium level below this threshold. An additional interesting result that raises questions about the induction mechanism of iLTP is that the magnitude was reduced with spiking from −70 mV (as compared with from −60 mV). This seems surprising if iLTP is solely determined by the amount of calcium entering through L-type channels. Although the proportion of calcium coming through L-type channels is lower with spiking from −70 mV, it might be expected that identical amounts of L-type-mediated calcium entry would occur with spiking from both membrane potentials, but that the proportion is changed due to a greater amount of calcium entering through T-type channels with spiking from −70 mV. Also, it is interesting to note that pairing in the presence of nimodipine, where presumably the total calcium entry would be through T-type channels, appeared to persistently depress IPSPs. Perhaps taken together, these results hint at a more complex induction pathway such as where calcium entry via L-type channels increases iLTP magnitude while calcium that enters via T-type channels decreases iLTP.

The ability of LTCPs to trigger iLTP makes it reasonable to speculate physiological induction of this form of plasticity may occur during sleep oscillations seen in vivo, and the subsequent calcium entry that occurs (Dossi et al. 1992; Errington et al. 2012). Sieber et al. (2013) make this connection in part due to the frequency dependence of iLTP induction as it corresponds to the low-frequency rates of firing that occur during sleep. While intriguing, given that Sieber et al. did not control for the duration of spiking and simply applied the same number of spikes at different frequencies, it is also possible that instead the key factor is the duration of raised calcium concentration. Furthermore, it is not reported whether neurons are in fact able to burst at 5 Hz.

If the plasticity outlined by Sieber et al. (2013) were to occur in vivo during sleep, we would expect to see an increase in inhibition from the TRN. This could subsequently enhance the generation of sleep spindles, as these are dependent on inhibitory drive from the TRN (Contreras and Steriade 1996; Fuentealba and Steriade 2005). Taken together, these results in conjunction with others (e.g., Hsu et al. 2010; Castro-Alamancos and Calcagnotto 1999) show that the adult thalamus is more than just a passive relay of external information; it is, in fact, a dynamic distributor capable of regulating its outputs depending on the previous input and firing history.

## GRANTS

S. R. Hulme is supported by Alzheimers Research UK Grant ARUK-NCG21013A-2, and W. M. Connelly is supported by Wellcome Trust Grant 91882. The funders took no part in the writing of the manuscript.

## DISCLOSURES

No conflicts of interest, financial or otherwise, are declared by the author(s).

## AUTHOR CONTRIBUTIONS

S.R.H. and W.M.C. drafted manuscript; S.R.H. and W.M.C. edited and revised manuscript; S.R.H. and W.M.C. approved final version of manuscript; W.M.C. prepared figures.
